# Low seroprevalence of hepatitis delta virus co-infection in hepatitis B virus-infected blood donors in China: A multicenter study

**DOI:** 10.3389/fmicb.2022.992817

**Published:** 2022-11-14

**Authors:** Le Chang, Ying Yan, Huimin Ji, Huizhen Sun, Xinyi Jiang, Zhuoqun Lu, Lunan Wang, HBV-Infected Blood Donors Study Group

**Affiliations:** ^1^National Center for Clinical Laboratories, Institute of Geriatric Medicine, Chinese Academy of Medical Sciences, Beijing Hospital/National Center of Gerontology, Beijing, China; ^2^Beijing Engineering Research Center of Laboratory Medicine, Beijing Hospital, Beijing, China; ^3^National Center for Clinical Laboratories, Peking Union Medical College, Chinese Academy of Medical Sciences, Beijing, China

**Keywords:** hepatitis delta virus, seroprevalence, blood donors, hepatitis B virus surface antigen, hepatitis B virus

## Abstract

Hepatitis delta virus (HDV) coinfected with HBV causes severe viral hepatitis, however, the number of HDV infection may be underestimated. In the present study, we enrolled 1,141,331 blood donations, routinely tested for HBsAg and/or HBV DNA, from 21 blood establishments in China. 2,690 donors were HBsAg and/or HBV DNA positive after screening tests. After verification of HBsAg and HBV DNA, 1,490 samples were HBsAg confirmed-positive, including 1,459 HBV DNA-positive samples, and 825 samples were seronegative but HBV DNA positive. We first analyzed demographic characteristics of involved 2,690 donors with different HBV infection status and found the proportions of males, the older donors, workers and farmers were higher in HBsAg-/HBV DNA+ group. Then we evaluated specificity of HDV IgG and IgM antibody assays with 375 HBsAg and HBV DNA confirmed-negative samples, and 374 were tested negative using the two assays, respectively, suggesting a specificity of 99.73% for both assays (374/375, 95% Cl: 98.51–99.95%). Subsequently, we tested for HDV IgG and IgM of 2,315 HBsAg and/or HBV DNA confirmed-positive samples, and nine showed reactivity for IgG, while two were reactive for IgM. All these 11 reactive samples were tested again with another HDV pan-Ig and IgM testing assays and HDV RNA, and only one donor was identified as HDV IgG positive and HDV RNA negative, showing an HDV seroprevalence of 0.067% (95%CI: 0.012–0.38%) among HBsAg-positive blood donors in China. The positive donor was followed up for 2 years after the donation date, and decreased antibody titer of HDV IgG and HBsAg conversion were observed, and the infection status of the donor was HDV infection with recovery and occult hepatitis B virus infection with genotype C2. These results indicated a low seroprevalence of HDV infection among blood donors and a low risk of HDV transmission through blood transfusion in China.

## Introduction

Hepatitis delta virus (HDV) is a satellite RNA virus that depends on the hepatitis B virus (HBV) for its life cycle (Hughes et al., 2011). Chronic HBV infection is an important cause of liver-related morbidities. Globally, 257 million people live with chronic HBV infection (WHO, 2017), and HBV coinfection with HDV usually leads to more severe viral hepatitis, or a more frequent fulminant course of hepatitis (Miao et al., 2022). Previous meta-analysis studies estimated that 0.16–1.07% of the global general population, and 4.5–14.57% of HBsAg-positive individuals, have antibodies against HDV (anti-HDV) (Chen et al., 2019; Miao et al., 2020; Stockdale et al., 2020; Shen et al., 2021).

Currently, the tested rates of HDV among HBV-infected populations are low. A nationwide retrospective study of veterans in the U.S. from October 1999 to December 2013 included 25,603 HBsAg-positive patients, and only 8.5% were once tested for HDV (Kushner et al., 2015). Similarly, only 1.6% of patients in China tested anti-HDV antibodies or related biomarkers among 832,144 HBsAg-positive cases over the past 10 years (Liu et al., 2022). Although coinfection with HDV and HBV has attracted increasing attention, only 62% of HBV patients were tested for HDV in London from 2005 to 2012, indicating inadequate screening for HDV in HBV patients (El Bouzidi et al., 2015). This suggests that the global prevalence of HDV may be underestimated.

Although HDV is a blood-borne virus, the rate of HDV infection in blood donors is generally not known. In Iran and France, the seroprevalence of HDV in HBsAg-positive blood donors was between 1.98 and 2.0% (Attaran et al., 2014; Servant-Delmas et al., 2014). However, 19.78% of HBsAg-positive blood donors were coinfected with HDV in Mauritania (Mansour et al., 2012). To date, there have been no large-scale multicenter studies of the rate of HDV infection in blood donors in China. In the present study, we involved 21 blood centers from 14 provinces or municipalities in China and tested the anti-HDV rate of 1,490 HBsAg-positive donation samples collected from March 2020 to January 2021. This is the first study to evaluate the prevalence of HDV among blood donors in China.

## Materials and methods

### Study design and participants

**Figure 1:**
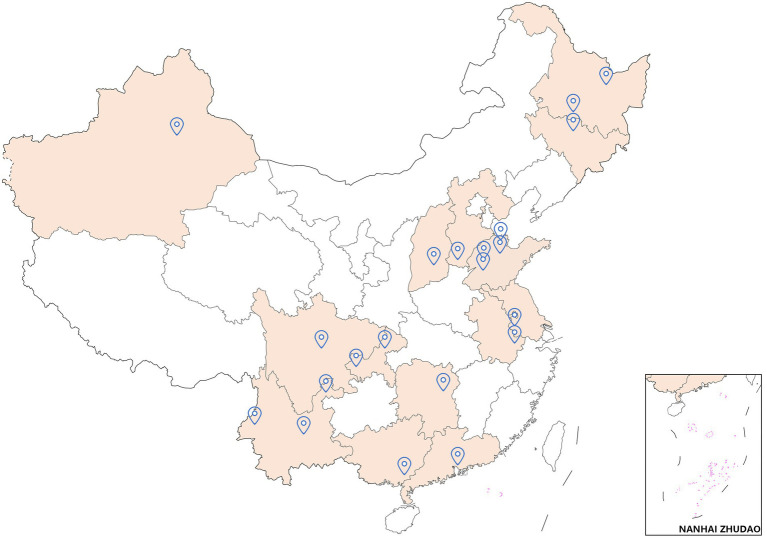
The distribution of blood centers involved in the study. 21 blood centers from 14 provinces or municipalities in China were enrolled in the present study (red colored regions and blue position marks in the map). The list of enrolled blood centers is in Supplementary Table S1.

Blood donation samples collected from March 2020 to January 2021 from 21 blood centers in 14 provinces or municipalities in China were enrolled in the present study. The distribution of blood centers is shown in Figure 1. Usually, a series of pre-donation tests are performed before blood collection, including HBsAg and alanine transaminase (ALT) rapid testing. Post-donation tests included HBsAg, anti-HCV, anti-HIV, and anti-Treponema pallidum (TP) antibodies using two different enzyme-linked immunosorbent assay (ELISA) assays, nucleic acid test (NAT, HBV DNA, HCV RNA, and HIV RNA) and ALT, and were performed at blood screening laboratories. All available plasma samples with HBsAg reactivity and/or HBV DNA positivity were collected from discarded plasma bags and sent to the National Center for Clinical Laboratories for verification and further testing. Anonymous personal demographic information was collected from blood donors, including sex, age, ethnicity, occupation, and blood type.

### Validation of HBsAg and hepatitis B virus DNA

**Figure 2:**
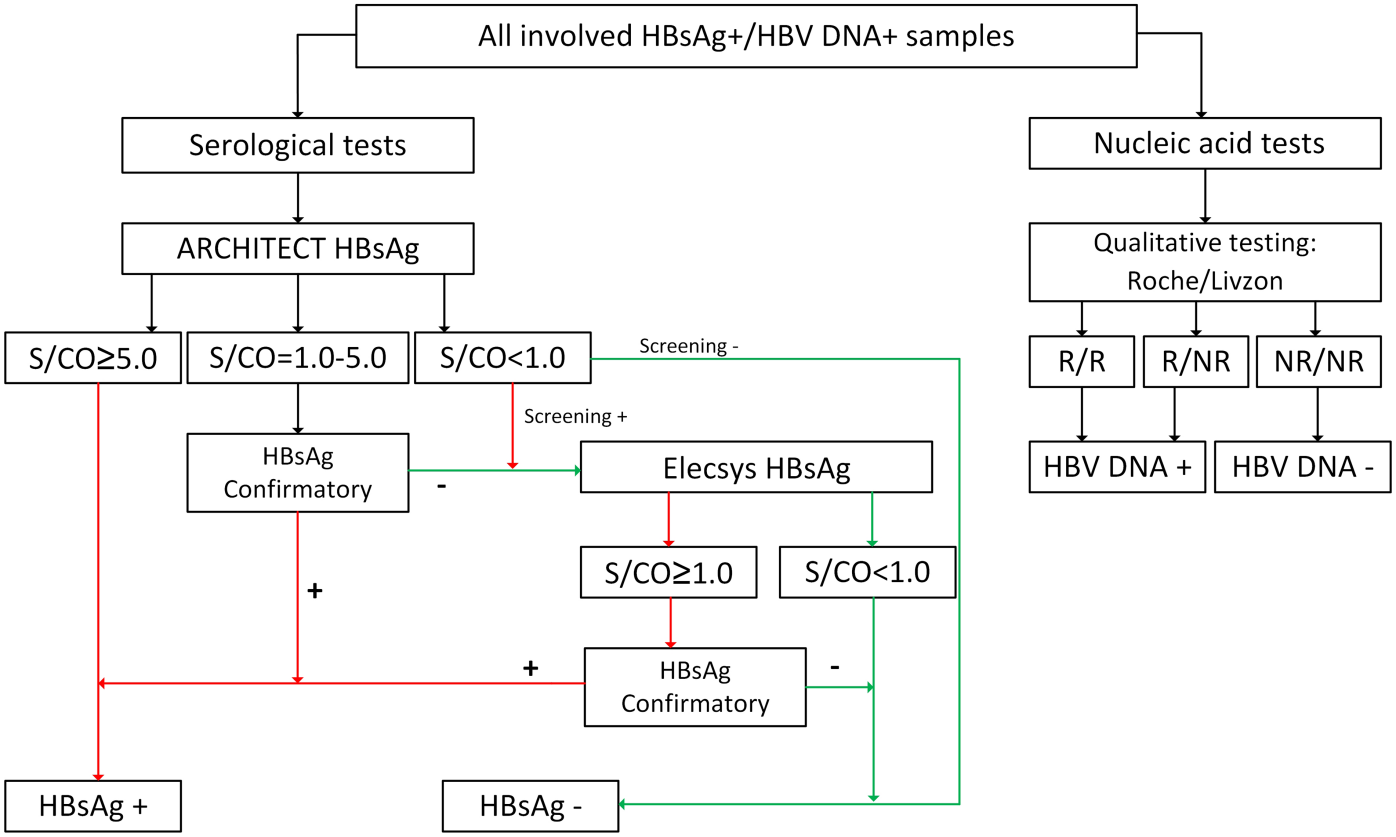
Confirmatory process of HBsAg and HBV DNA. +, reactive/positive; −, nonreactive/negative; R, reactive; NR, nonreactive; ARCHITECT HBsAg, ARCHITECT HBsAg Qualitative II (Abbott Ireland Diagnostics Division, Sligo, Ireland); Elecsys HBsAg, Elecsys HBsAg II (Roche Diagnostics GmbH, Mannheim, Germany). HBsAg Confirmatory, ARCHITECT HBsAg Qualitative II Confirmatory (Abbott Ireland Diagnostics Division), or HBsAg Confirmatory Test (Roche Diagnostics GmbH). Roche, cobas TaqScreen MPX Text, version 2.0 (Roche Molecular Systems, Inc., Branchburg, NJ, United States); Livzon, HBV DNA/HCV RNA/HIV-(1 + 2) RNA Diagnostic Kit (PCR- Fluorescence Probing) (Livzon Diagnostics, Zhuhai, China).

All HBsAg-reactive and/or HBV DNA-positive blood samples were further tested for HBsAg using the ARCHITECT HBsAg Qualitative II assay (Abbott Ireland Diagnostics Division, Sligo, Ireland; LOD: 0.05 IU/mL), and the reactive samples with signal to cutoff (S/CO) values from 1.0 to 5.0 were validated using the ARCHITECT HBsAg Qualitative II Confirmatory assay (Abbott Ireland Diagnostics Division). Samples that were HBsAg negative but screened as HBsAg reactive were tested again using the Elecsys HBsAg II assay (Roche Diagnostics GmbH, Mannheim, Germany; LOD: 0.05 IU/mL) and the reactive samples were further confirmed using an HBsAg Confirmatory Test assay (Roche Diagnostics GmbH). Qualitative nucleic acid tests were performed using the Cobas TaqScreen MPX Test, version 2.0 (Roche Molecular Systems, Inc., Branchburg, NJ, United States; LOD: 2.3 IU/mL) and HBV DNA/HCV RNA/HIV-(1 + 2) RNA Diagnostic Kit (PCR- Fluorescence Probing) (Livzon Diagnostics, Zhuhai, China; LOD: 5 IU/mL). The processes used to confirm HBsAg and HBV DNA positivity are summarized in Figure 2. In addition, HDV-positive blood donors were followed up and further tested for other HBV markers, including anti-HBs (Abbott Ireland Diagnostics Division), HBeAg, anti-HBe, anti-HBc, and anti-HBc IgM (Abbott GmbH, Wiesbaden, Germany), and the HBV viral load was determined using the COBAS AmpliPrep/COBAS Taqman HBV Test, version 2.0 (Roche Molecular Systems, Inc., Branchburg, NJ, United States; LOD: 9 IU/mL).

### Serological tests for hepatitis delta virus

Hepatitis delta virus IgG and IgM antibodies were detected using HDV IgG and IgM testing kits (Wantai BioPharm, Beijing, China). To avoid potential false reactivity caused by nonspecific binding antibodies, HDV IgG- or IgM-reactive samples were further tested for HDV pan-Ig and IgM using two additional testing kits (Dia.Pro Diagnostic Bioprobes, S.r.l., Sesto San Giovanni, Italy). All four serological tests were performed by ELISA according to the manufacturer’s instructions, as reported previously (Brichler et al., 2014; Aberra et al., 2018; Besombes et al., 2020). In brief, IgG antibodies were tested using an indirect ELISA method with recombinant HDV antigen (HDAg). The two IgM antibody tests were based on the μ-chain capture method with recombinant HDAg. Pan-Ig detection was based on a competitive immunoassay using recombinant HDAg. A signal to cutoff value (S/CO) of ≥1.0 was defined as reactive.

### Hepatitis delta virus nucleic acid test

Hepatitis delta virus antibody-reactive samples were tested for HDV RNA using the AccuVL HDV assay (Shanghai Haoyuan Biotech, Shanghai, China), which targets the HDAg ORF region of the viral genome. Nucleic acids were extracted from 1,200 μL of plasma. A cycle threshold (Ct) value of ≤40 was defined as a positive result. HDV genotypes 1–8 all could be detected. The limit of detection of the assay was 5 IU/mL. A positive control based on the pseudovirus containing the whole genome of HDV (GenBank: D01075.1) and a negative control (no HDV template) were included in each experimental batch.

### Genotyping and sequencing of hepatitis B virus

For HDV-antibody-confirmed positive donors, HBV DNA was extracted from 1.2 mL of plasma, and the preS/S region was amplified by nested PCR, followed by Sanger sequencing, which was performed by Tsingke Biotechnology Co., Ltd. The first-round PCR reaction was performed in 25 μL reaction volumes containing 15 μL of DNA, 0.2 mM of dNTP mix, 0.2 μM of each primer, and 0.625 U of Amplitaq DNA polymerase. The second-round reaction was performed in 25 μL reaction volumes containing 2 μL of first-round products, 0.2 mM of dNTP mix, 0.2 μM of each primer, and 0.625 U of Amplitaq DNA polymerase. The first-round amplification program was: 95°C for 2 min, 40 cycles of 95°C for 15 s, 55°C (S region) or 53.5°C (preS region) for 30 s, and 72°C for 1 min 30 s (S region) or 1 min (preS region), and 72°C for 10 min. The second-round amplification program was: 95°C for 2 min, 35 cycles of 95°C for 15 s, 55°C (S region) or 53.5°C (preS region) for 30 s, 72°C for 1 min, and 72°C for 10 min. Nested PCR and sequencing were performed using the primers listed in Supplementary Table S1.

### Statistical analysis

Differences in demographic characteristics among HBsAg+, HBsAg-/HBV+, and HBsAg-/HBV DNA-, blood donors were estimated using the chi-square test. The specificities of the HDV IgG and IgM assays were calculated as the number of tested negative samples according to the two assays divided by the total number of tested confirmed-negative samples. The 95% confidence intervals (CIs) of the specificity evaluation and HDV seroprevalence were calculated using OpenEpi, Version 3 (Dean et al., 2013). Statistical significance was set at <0.05. All data were collected in Microsoft Excel 365 (Microsoft Corporation by Impressa Systems, Santa Rosa, CA, United States) and statistical analysis was performed using SPSS v21.0 (IBM SPSS, Chicago, IL, United States).

## Results

### Validation of HBsAg and hepatitis B virus DNA

**Figure 3:**
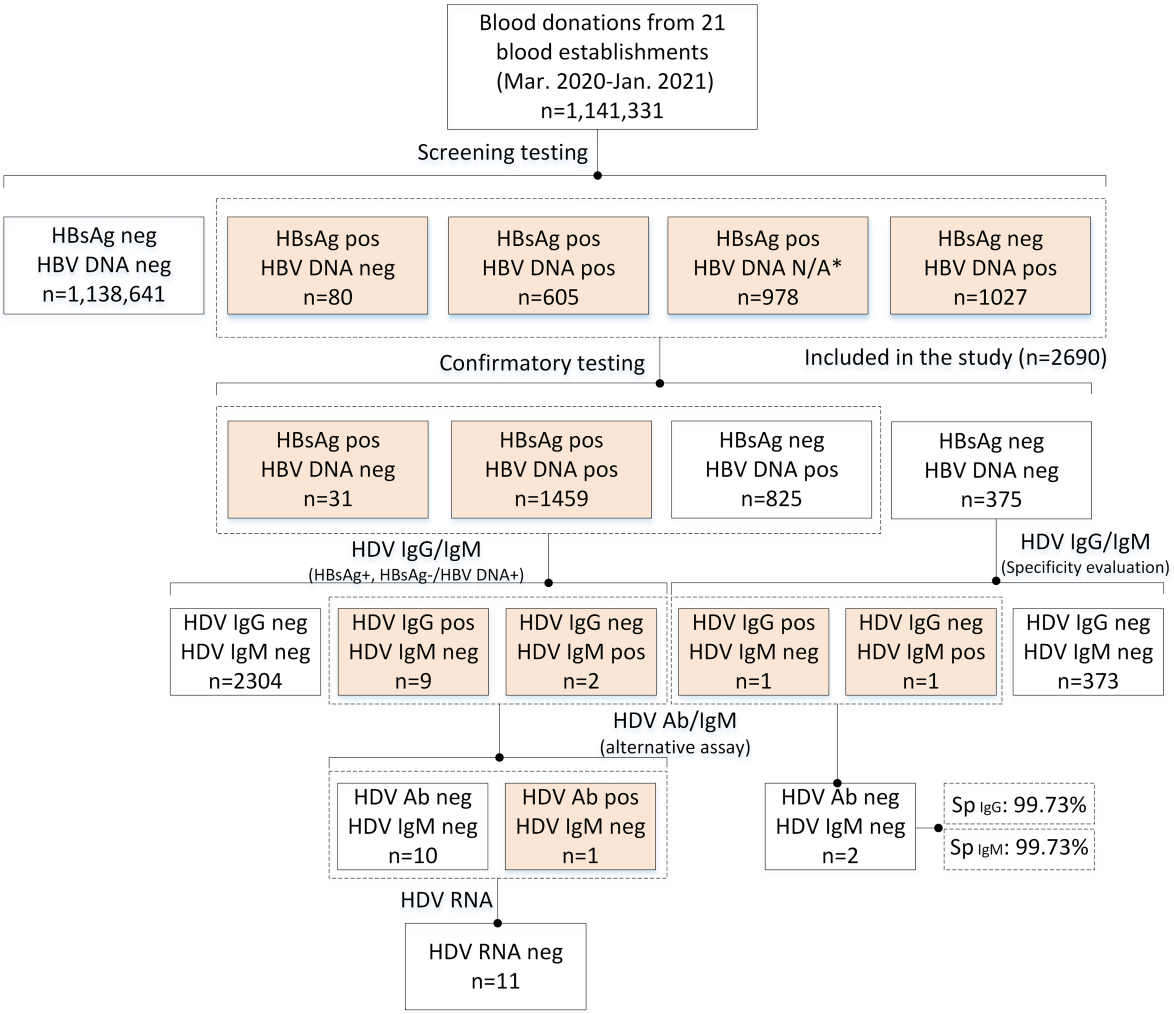
Flow chart of the study. Pos/+, positive/reactive; neg/−, negative/nonreactive; N/A, unavailable.

A total of 1,141,331 blood donation samples collected by 21 blood establishments in 14 provinces or municipalities in China, between March 2020 and January 2021, were screened (Figure 3). Out of these, 2,690 available samples were HBsAg screening reactive and/or HBV DNA positive and were enrolled in the present study. Finally, 1,490 samples were confirmed as HBsAg positive, including 31 HBV DNA-negative and 1,459 HBV DNA-positive samples. A total of 1,200 donations were HBsAg-negative, including 375 HBV DNA-negative samples, which were used for specificity evaluation of the HDV IgG/IgM screening assays. The number of samples screened at each blood center is shown in Supplementary Table S2.

### Characteristics of enrolled donors

After verification of HBsAg and HBV DNA, 2,690 donors were included in the HDV tests, and the characteristics of enrolled donors are summarized in Table 1. Of the donors tested for HDV, 68.6% were male, and the median age was 42 years (IQR: 33–48). We further analyzed the demographic information of the HBsAg-positive, HBsAg-/HBV DNA+, and HBsAg-/HBV DNA- groups. The proportion of males was higher in the HBV-infected groups (*p* = 0.000003), especially in the HBsAg-/HBV DNA+ group, of which 79.5% were male. Donors from the HBsAg-/HBV DNA+ group also tended to be older than those from the other two groups (*p* < 0.000001). In terms of occupation, the proportion of students and freelancers was lower in the HBsAg-/HBV DNA+ group, while that of workers and farmers was higher (*p* < 0.000001). ABO blood type and ethnicity showed no differences among the three groups (*p* = 0.417804, and *p* = 0.239999, respectively).

**Table 1:** Characteristics of enrolled donors (*n* = 2,690).

	**All**		**HBsAg+**		**HBsAg-/DNA+**		**HBsAg-/DNA-**		***p*-value**
	**(*n* = 2,690)**		**(*n* = 1,490)**		**(*n* = 825)**		**(*n* = 375)**		
**Sex (%)**									3E-06
Male	1,845	(68.6)	1,036	(69.5)	593	(71.9)	216	(57.6)	
Female	845	(31.4)	454	(30.5)	232	(28.1)	159	(42.4)	
**Age, median (IQR)**	42 (33–48)		40 (31–47)		45 (37–50)		43 (33–49)		<0.000001
18–25	320	(11.9)	232	(15.6)	55	(6.7)	33	(8.8)	
26–35	550	(20.4)	324	(21.7)	133	(16.1)	93	(24.8)	
36–45	762	(28.3)	435	(29.2)	242	(29.3)	85	(22.7)	
46–55	1,027	(38.2)	497	(33.4)	373	(45.2)	157	(41.9)	
>55	31	(1.2)	2	(0.1)	22	(2.7)	7	(1.9)	
**ABO blood type (%)**									0.4178
A	852	(31.7)	482	(32.3)	261	(31.6)	109	(29.1)	
B	216	(8)	121	(8.1)	63	(7.6)	32	(8.5)	
O	675	(25.1)	384	(25.8)	207	(25.1)	84	(22.4)	
AB	947	(35.2)	503	(33.8)	294	(35.6)	150	(40)	
**Ethnicity (%)**									0.24
Han	2,577	(95.8)	1,420	(95.3)	800	(97)	357	(95.2)	
Non-Han	107	(4)	66	(4.4)	23	(2.8)	18	(4.8)	
Missing data	6	(0.2)	4	(0.3)	2	(0.2)	0	(0)	
**Occupation (%)**									<0.000001
Student	141	(5.2)	110	(7.4)	20	(2.4)	11	(2.9)	
Freelancer	244	(9.1)	154	(10.3)	44	(5.3)	46	(12.3)	
Office worker	323	(12)	163	(10.9)	110	(13.3)	50	(13.3)	
Worker	308	(11.4)	151	(10.1)	120	(14.5)	37	(9.9)	
Business and service personnel	51	(1.9)	31	(2.1)	15	(1.8)	5	(1.3)	
Civil worker /teacher/healthcare worker	133	(4.9)	44	(3)	57	(6.9)	32	(8.5)	
Farmer	374	(13.9)	188	(12.6)	135	(16.4)	51	(13.6)	
Military personnel	6	(0.2)	2	(0.1)	2	(0.2)	2	(0.5)	
Others	921	(34.2)	559	(37.5)	249	(30.2)	113	(30.1)	
Missing data	189	(7	88	(5.9)	73	(8.8)	28	(7.5)	

### Specificity evaluation of hepatitis delta virus assays

A total of 375 HBsAg-/HBV DNA- blood samples were tested for HDV IgG and IgM antibodies. 373 samples were anti-HDV-negative, while one was HDV-IgG reactive, and the other was HDV IgM reactive. Because HDV coinfection with other non-HBV viruses is rare, further tests were performed to exclude potential false reactivity. Negative results for these two samples were observed using two additional HDV antibody assays (HDV pan-Ig and IgM testing kits), indicating false reactivity in the primary tests and highlighting the necessity of supplementary anti-HDV tests. We then calculated the specificity of the HDV IgG, and IgM testing kits used in the primary tests, which were 99.73% for both tests (374/375, 95% Cl: 98.51–99.95%).

### Seroprevalence of hepatitis delta virus among hepatitis B virus-infected donors

A total of 1,490 samples from HBsAg-positive donors and 825 HBsAg-/HBV DNA+ samples were tested for HDV antibodies (IgG and IgM). Eleven donors showed reactivity: nine were HDV-IgG-positive and two were IgM-positive. Alternative tests were performed on these samples, and only one HBsAg-positive sample was HDV-pan-Ig-positive, suggesting a low seroprevalence for HDV of 0.067% (1/1,490, 95%Cl: 0.012–0.38%) among HBsAg-positive donors. In addition, we tested for HDV RNA in all 11 anti-HDV-reactive samples, and all showed negative results, indicating that the HDV-positive donor (no. B13 200014) was a previous HDV infection. The results of the anti-HDV screening-reactive donors are shown in Table 2.

**Table 2:** Results of anti-HDV and HDV RNA of anti-HDV screening-reactive donors.

Group	No. of each donor	Primary tests				Alternative tests				HDV RNA
		HDV IgG		HDV IgM		HDV pan-Ig		HDV IgM		
		S/CO	Result	S/CO	Result	S/CO	Result	S/CO	Result	Result
HBsAg+	B13 200014	25.32	R	0.56	N	7.38	R	0.24	N	N
HBsAg+	B35 200140	2.71	R	0.31	N	0.29	N	0.24	N	N
HBsAg+	B35 200148	2.21	R	0.22	N	0.27	N	0.23	N	N
HBsAg+	B50 200215	1.66	R	0.29	N	0.25	N	0.27	N	N
HBsAg+	B42 200022	2.46	R	0.07	N	0.17	N	0.27	N	N
HBsAg+	B46 200390	1.26	R	0.11	N	0.25	N	0.21	N	N
HBsAg+	B35 200068	0.04	N	2.68	R	0.34	N	0.26	N	N
HBsAg+	B13 200382	0.07	N	1.21	R	0.25	N	0.2	N	N
HBsAg-/HBV DNA+	B14 200002	2.76	R	0.06	N	0.3	N	0.23	N	N
HBsAg-/HBV DNA+	B13 200094	1.56	R	0.14	N	0.23	N	0.23	N	N
HBsAg-/HBV DNA+	B13 200095	1.9	R	0.13	N	0.21	N	0.24	N	N

S/CO, signal to cutoff value; R, reactive; N, nonreactive.

### Follow-up of anti-HDV positive donor

Because only one anti-HDV-positive donor (no. B13 200014) was found, we followed up the donor and tested HBV- and HDV-related markers for 2 years after the donation date (Table 3). After 641 days, HBsAg was negative whereas other HBV markers (HBsAb, HBeAg, HBeAb, and HBcAb) remained stable. HDV IgG was persistently positive for 761 days, but the S/CO value decreased gradually. These results indicate that the infection status of the donor was HDV infection with recovery and occult HBV infection (OBI). The HBV preS/S regions of the three follow-up samples were sequenced and genotyped as C2.

**Table 3:** Follow-up results of an anti-HDV-positive donor.

	No.	B13 200014	B13 200014a	B13 200014b
HBVmarkers	Collection date	2020-4-14	2022-1-15	2022-5-15
	Days since donation	0 day	641 days	761 days
	HBsAg (S/CO)-ARCHITECT	R (4.88)	NR (0.400)	NR (0.430)
	HBsAg (S/CO)-Elecsys	R (4.01)	NR (0.735)	NR (0.649)
	HBsAb (mIU/mL)	0	0	0
	HBeAg	NR	NR	NR
	HBeAb	R	R	R
	HBcAb	R	R	R
	HBcAb IgM	NR	NR	NR
	HBV DNA (Qualitive, Ct value)	33.5	33.7	34.2
	HBV viral load (IU/mL)	< 20	32.6	< 20
	HBV genotype	C2	C2	C2
HDV markers	HDV IgG (S/CO)-Wantai	R (25.32)	R (17.12)	R (5.06)
	HDV IgM (S/CO)-Wantai	NR (0.562)	NR (0.500)	NR (0.500)
	HDV Ab (S/CO)-Dia.Pro	R (7.38)	R (6.77)	R (4.79)
	HDV IgM (S/CO)-Dia.Pro	NR (0.243)	NR (0.257)	NR (0.260)
	HDV RNA	NR	NR	NR

HBsAg-ARCHITECT, ARCHITECT HBsAg Qualitative II (Abbott Ireland Diagnostics Division, Sligo, Ireland); HBsAg (S/CO)-Elecsys, Elecsys HBsAg II (Roche Diagnostics GmbH, Mannheim, Germany); HDV IgG-Wantai, HDV IgG testing kit (Wantai BioPharm, Beijing, China); HDV IgM-Wantai, HDV IgM testing kit (Wantai BioPharm, Beijing, China); HDV Ab-Dia.Pro, HDV pan-Ig testing assay (Dia.Pro Diagnostic Bioprobes, S.r.l., Sesto San Giovanni, Italy); HDV IgM-Dia.Pro, HDV IgM testing assay (Dia.Pro Diagnostic Bioprobes, S.r.l., Sesto San Giovanni, Italy); R, reactive; NR, non-reactive.

## Discussion

Although there has recently been a resurgence in the awareness of hepatitis D, HDV remains neglected. HDV-related biomarkers testing is not routinely performed in patients with chronic hepatitis B in most countries, especially in HBV high-epidemic regions. Thus, the true prevalence of HDV may be far greater than the existing data suggests (Ahn and Gish, 2014). In the present multicenter study, we screened over 1 million blood donation samples across 21 blood centers in China and reported, for the first time, a low seroprevalence (0.067%, 1/1490) of HDV among HBsAg-positive blood donors, thus contributing important new data to the global HDV epidemiological map.

The global burden of HDV infection has recently been updated to more than 60 million cases (Chen et al., 2019; Miao et al., 2020). HDV is highly prevalent in Central and Western sub-Saharan Africa, Central Asia, Latin America, and Eastern Europe (Miao et al., 2020). As HDV is a defective virus, the prevalence of HDV is highly associated with the prevalence of HBV. China was historically one of the highly endemic countries for HBV; however, due to the widespread administration of the HBV vaccine in newborns since 1992, and improvements in health awareness and socio-economic conditions, the prevalence of HBV in China has decreased significantly (Goyal and Murray, 2017). The HDV prevalence in China is reported to be as low as 0.45% (95%CI: 0.15–0.89%) in the general population, which is similar to that of Japan, Albania, and Saudi Arabia (Chen et al., 2019). Similar to mainland China, Taiwan has also realized decreased seroprevalence of HDV since 2006, with seropositive rates of HDV progressively decreasing from 18.6 to 3.7% among HBsAg-positive patients (Lee et al., 2020). Despite its low seroprevalence, China still has a largest burden of HDV infection, representing 8.68% of the total global disease burden, due to its large population base (Goyal and Murray, 2017).

As it is a transfusion-transmitted pathogen, HDV-infected blood donors could threaten blood safety. In the present study, only one blood donor was positive for HDV antibodies, showing an extremely low seroprevalence of HDV infection among all blood donors (0.000088%, 1/1,141,331) and HBsAg-positive donors (0.067%), which was lower than the existing rates for blood donors from other countries (Mansour et al., 2012; Attaran et al., 2014; Uzun et al., 2014; Juhl et al., 2020). This result also suggests that the risk of HDV transmission through blood transfusions is very low in China. There are several factors that may have led to this low prevalence. First, the population of blood donors usually comprises healthy individuals; thus, the prevalence of HBV or other transfusion-transmitted pathogens is lower among blood donors than in the general population. Data from the present study also indicates that the positive rate of screened HBsAg-positive and/or HBV DNA-positive donors among all 1,141,331 blood donors is only 0.24% (2,690/1,141,331, 95%CI: 0.227–0.245%), which is far lower than the 8.97% HBsAg positive rate among the general population aged 20–59 years who were surveyed in China in 2006 (Liang et al., 2009). Second, in China, a series of pre-donation rapid tests are routinely performed before blood collection, including HBsAg and ALT. Therefore, individuals with elevated ALT (≥50 U/L) and HBV-infected individuals may be prevented from donating blood. Third, the participants enrolled in the present study were only from 14 provinces, and may not be representative of the whole of China. A previous study showed that HDV seroprevalence among HBsAg-positive patients in China is limited to geographic hotspots, such as Inner Mongolia (35/251, 13.9%) and Xinjiang (7/180, 3.9%) (Roggenbach et al., 2021). In the present study, we did not collect samples from blood donors in Inner Mongolia, and the number of confirmed HBsAg-positive donors from Xinjiang was only 58, which suggests that HDV-infected blood donors from these hotspot regions may not have been included in the present study. In addition, all the tested donations were collected from plasma bags that contained approximately 14% preservative fluid, which did not affect the detection of HDV antibodies but might dilute the samples and lower the sensitivity of the assays.

Antibody testing is often used as the primary screening test for HDV infections (Chen et al., 2021). IgM antibodies against HDAg are detectable approximately 4 weeks after exposure, and decrease and gradually disappear at 2 months after acute infection, or can be elevated in patients with chronic HDV during disease progression. In addition, HDV IgM can persist for a long time in cases of HDV superinfection. Anti-HDV IgG can persist for a long time after viral clearance, making it difficult to distinguish between present and previous HDV infections. HDV RNA testing is a useful supplement to compensate for the shortcomings of HDV antibody detection. The detection of RNA usually implies the presence of active HDV infection, but many RNA-based tests had limited genotype coverage, leading to concerns of false negative results (Ahn and Gish, 2014; Brichler et al., 2013, 2014). In addition, since the specificity of HDV antibody assays is not 100% (99.73% in the present study), for HDV antibody-reactive and HDV RNA-negative samples, it is necessary to test specific antibodies again using a different assay to avoid potential false positive results, especially in HDV non-endemic areas. Thus, in the present study, we used different antibody testing assays from different manufacturers combined with an HDV RNA test that covered all eight HDV genotypes to reflect the true HDV infection status among blood donors in China. As a result, we found 11 blood donors who were positive for HDV antibodies according to the anti-HDV screening tests. Although these donors were all HDV RNA-negative at the time of donation, the level of viremia may fluctuate over time (Schaper et al., 2010), and periodic testing for HDV RNA is therefore required. In addition, to ensure the safety of blood for transfusion, HDV-RNA testing of blood donors is recommended in highly endemic areas.

HDV is usually co-infected or super-infected with HBV, although a few studies have reported that HCV can also help to disseminate HDV in the absence of HBV (Perez-Vargas et al., 2019). HDV typically relies on surface glycoproteins (GPs) from HBV for virion assembly, envelopment, and cellular transmission, and does not encode envelope proteins for packaging of its ribonucleoprotein (RNP). Thus, active HDV infection is usually accompanied by plasma HBsAg positivity. Therefore, the AASLD 2018 Hepatitis B Guidance recommends testing HBsAg-positive individuals who are at risk for HDV (Terrault et al., 2018). However, covert HDV infection could also occur in HBsAg-negative but HBV DNA-positive (designated as OBI) donors. In the present study, we found a donor with previous HDV infection, who achieved HBsAg conversion during the subsequent 2 years, highlighting the existence of an OBI population, although rare, who would not need to be routinely tested for HDV (Delfino et al., 2012).

In conclusion, we first screened HBV markers in 1,141,331 donation samples collected from 21 blood centers located in 14 provinces in China. We found only one HDV antibody-positive among 1,490 HBsAg-positive donors, with an extremely low seroprevalence of 0.067% (95%CI: 0.012–0.38%) and no current HDV infections. These results indicate a low risk of HDV transmission *via* blood transfusion in China, although further investigations are required in Chinese provinces with high HDV seroprevalence.

## Data availability statement

The raw data supporting the conclusions of this article will be made available by the authors, without undue reservation.

## Ethics statement

The studies involving human participants were reviewed and approved by Medical Ethical Committee of Beijing Hospital. The patients/participants provided their written informed consent to participate in this study. Written informed consent was obtained from the individual(s) for the publication of any potentially identifiable images or data included in this article.

## Author contributions

LC and LWa: conceptualization and methodology. LC and ZL: data curation. LC, YY, HS, and XJ: formal analysis. LWa and HBV-Infected Blood Donors Study Group: funding acquisition. LC, HJ, ZL, and HBV-Infected Blood Donors Study Group: investigation. LWa: project administration, resources, supervision, and writing – review and editing. LC, YY, HJ, and ZL: validation. LC and YY: visualization. LC: writing – original draft. All authors contributed to the article and approved the submitted version.

## Funding

This work was supported by the CAMS Innovation Fund for Medical Sciences (grant number 2021-I2M-1-060; LWa) and Scientific-Health Joint Medical Research Project of Chongqing (grant number 2022MSXM093; LWe).

## Conflict of interest

The authors declare that the research was conducted in the absence of any commercial or financial relationships that could be construed as a potential conflict of interest.

## Publisher’s note

All claims expressed in this article are solely those of the authors and do not necessarily represent those of their affiliated organizations, or those of the publisher, the editors and the reviewers. Any product that may be evaluated in this article, or claim that may be made by its manufacturer, is not guaranteed or endorsed by the publisher.
