# Supplementary material for: Low seroprevalence of hepatitis delta virus co-infection in hepatitis B virus-infected blood donors in China: A multicenter study
Source: Front Microbiol. 2022 Nov 14;13:992817. doi: 10.3389/fmicb.2022.992817 (PMC9702509; doi:10.3389/fmicb.2022.992817)
Supplement: Supplementary file 1 [file Table_1.pdf]

Supplementary material Table S1

Low seroprevalence of hepatitis delta virus co-infection in hepatitis B virus-infected blood donors in China: a multicenter study

Le Chang, Ying Yan, Huimin Ji, Huizhen Sun, Xinyi Jiang, Zhuoqun Lu, Lunan Wang, HBV-Infected Blood Donors Study Group

Correspondence: lunan99@163.com; Tel.: +86 10 85133609

Table S1. Primers for nested-PCR and sequencing of HBV

**preS region**	**Primers**	**Sequence (5'-3')**
First round	HBV-2804F	GCCTCATTTTGYGGGTCACCAT
	HBV-668R	CTGAGGCCCACTCCCATAG
Second round /sequencing	HBV-2833F	GGGAACAAGAGCTACAGCATGG
	HBV-309R	GGTTGGGGACTGCGAATTT
**S region**	**Primers**	**Sequence (5'-3')**
First round	HBV-56F	CCTGCTGGTGGCTCCAGTTC
	HBV-1253R	GCAGTATGGATCGGCAGAGGAG
Second round /sequencing	HBV-178F	CCTAGGACCCCTGCTCGTGTTACAGGC
	HBV 1186R	CCAGTGGGGGTTGCRTCAGC
