# Supplementary material for: Low seroprevalence of hepatitis delta virus co-infection in hepatitis B virus-infected blood donors in China: A multicenter study
Source: Front Microbiol. 2022 Nov 14;13:992817. doi: 10.3389/fmicb.2022.992817 (PMC9702509; doi:10.3389/fmicb.2022.992817)
Supplement: Supplementary file 2 [file Table_2.pdf]

Supplementary material Table S2

Low seroprevalence of hepatitis delta virus co-infection in hepatitis B virus-infected blood donors in China: a multicenter study

Le Chang, Ying Yan, Huimin Ji, Huizhen Sun, Xinyi Jiang, Zhuoqun Lu, Lunan Wang, HBV-Infected Blood Donors Study Group

Correspondence: lunan99@163.com; Tel.: +86 10 85133609

Table S2. Number of donation samples screened in each blood center

Blood centers	Location (City, Province)	Donation date	Number of tested blood donations	HBsAg/HBV DNA screening positive for further testing	HBsAg confirmed positive	HBV DNA confirmed positive
Handan Central Blood Station	Handan, Hebei	2020.4-2020.9	58,294	89	47	58
Blood Center of Shandong Province	Jinan, Shandong	2020.2-2020.9	73,175	72	39	70
Zibo Blood Center	Zibo, Shandong	2020.3-2020.9	36,944	12	1	12
Dongying Central Blood Station	Dongying, Shandong	2020.4-2020.9	15,962	31	12	25
Tai'an Central Blood Station	Taian, Shandong	2020.4-2020.10	30,019	18	0	9
Central Blood Station of Changzhi	Changzhi, Shanxi	2020.4-2020.12	25,035	18	11	16
Jilin Provincial Blood Center	Changchun, Jilin	2020.3-2020.9	59,631	88	73	81
HarBin Red Cross Blood Center	Harbin, Heilongjiang	2020.4-2020.9	59,303	42	10	41
Jiamusi City Center Blood Station	Jiamusi, Heilongjiang	2020.4-2020.9	9,944	8	3	8
Changsha Blood Center	Changsha, Hunan	2020.4-2020.12	120,688	237	200	228
Shenzhen Blood Center	Shenzhen, Guangdong	2020.4-2020.9	62,997	165	72	134
Chengdu Blood Center	Chengdu, Sichuan	2020.4-2021.1	186,535	487	225	399
Xuancheng Blood Bank	Xuancheng, Anhui	2020.4-2020.12	16,674	52	39	52
Yulin Central Blood Station	Yulin, Guangxi	2020.4-2020.9	37,241	222	140	211
Nanjing Red Cross Blood Center	Nanjing, Jiangsu	2020.4-2020.9	47,904	86	29	59
Urumqi Blood Center	Urumqi, Xinjiang	2020.5-2020.12	35,204	99	58	69
Yunnan Kunming Blood Center	Kunming, Yunnan	2020.4-2020.9	87,696	144	95	134
Baoshan Central Blood Bank	Baoshan, Yunnan	2020.4-2020.9	9,077	9	5	7
Zhaotong Cental Blood Station	Zhaotong, Yunnan	2020.4-2020.0	19,460	45	25	41
Chongqing Blood Center	Chongqing	2020.4-2020.11	117,927	473	272	399

Chongqing Wanzhou Central Blood Station	Wanzhou District, Chongqing	2020.4-2020.9	31,621	293	134	231
Total			1,141,331	2,690	1,490	2,284
